# Supplementary material for: Estimating digital product trade through corporate revenue data
Source: Nat Commun. 2024 Jun 19;15:5262. doi: 10.1038/s41467-024-49141-z (PMC11186826; doi:10.1038/s41467-024-49141-z)
Supplement: Supplementary file 1 — Supplementary Information [file 41467_2024_49141_MOESM1_ESM.pdf]

Supplementary Information for:

Estimating Digital Product Trade through Corporate Revenue Data

Viktor Stojkoski[1,2], Philipp Koch[1,3], Eva Coll[1,4], César A. Hidalgo[1,5*]

[1]Center for Collective Learning, ANITI, IRIT, Université de Toulouse & CIAS Corvinus University of Budapest, Budapest, Hungary
[2]Faculty of Economics, University Ss. Cyril and Methodius, Skopje, North Macedonia
[3]EcoAustria – Institute for Economic Research, Vienna, Austria
[4]LEREPS, Sciences Po Toulouse, University of Toulouse Capitole, Toulouse, 31000 France

[5]Toulouse School of Economics and University of Toulouse Capitole, Toulouse, 31000, France

Table of Contents

Supplementary Note 1. Countries and digital sectors covered in the dataset 1

Supplementary Note 2. Digital revenue and consumption yearly summary statistics 5

Supplementary Note 3. Regression tree model features and cross-validation 7

Supplementary Note 4. Results with alternate allocation procedures for digital products consumption 11

Supplementary Note 5. Structure of digital products trade over the years 15

Supplementary Note 6. Concentration of digital products trade 16

Supplementary Note 7. Decoupling definitions and robustness checks 18

Supplementary Note 8. Economic complexity definitions, rankings, and regression analyses 25

Supplementary references 31

[*] Corresponding author email address: hidalgo.cesar@uni-corvinus.hu

Supplementary Note 1. Countries and digital sectors covered in the dataset

With our methodology we are able to provide estimates for bilateral trade in digital products between 189 countries and 31 digital sectors. Supplementary Table 1 lists the countries that are included in the dataset. Supplementary Table 2 gives the digital product sectors included in the dataset, provides short definition for their coverage, and maps them to the EBOPS and ISIC classifications. We point out that this mapping is only directional, as in practice, the reporting may not be accurately accounted for in many countries.[1]

Supplementary Table 1. List of digital product sectors included in the dataset.

Digital product sector	Description	EBOPS correspondence	ISIC correspondence
Cybersecurity	Cybersecurity services provide confidentiality, integrity, availability, and privacy of digital systems. These are measures for preventing and responding to cybercrimes, protecting computer systems, networks, programs, and data.	SI: Telecommunications, computer, and information services	6220 - Computer consultancy and computer facilities management activities
Mobile Application	A mobile application or app is a computer program or software application designed to run on a mobile device such as a phone, tablet, or watch.	SI: Telecommunications, computer, and information services	5829: Other software publishing
Cloud Computing	Cloud computing is the on-demand availability of computer system resources, especially computing power, without direct active management by the user	SI: Telecommunications, computer, and information services	6310 - Computing infrastructure, data processing, hosting, and related activities
File Hosting Service	File-hosting services allow users to upload files that can be accessed over the internet after providing an authentication key.	SI: Telecommunications, computer, and information services	6310 - Computing infrastructure, data processing, hosting, and related activities
Web Hosting	A web hosting service offers the facilities required for clients to create and maintain a site and makes it accessible on the World Wide Web.	SI: Telecommunications, computer, and information services	6310 - Computing infrastructure, data processing, hosting, and related activities
Data Licensing	Data licensing is the service of providing organized collection of data that can be stored and accessed electronically.	SH: Charges for the use of intellectual property n.i.e. / SI: Telecommunications, computer, and information services	6310 - Computing infrastructure, data processing, hosting and related activities
Digital Advertising	Digital Advertising is the use of the internet to deliver marketing messages via various formats to internet users. This includes advertisements displayed on search engines and social media, as well as video and banner advertising on specific websites.	SJ: Other business services	6390 - Web search portals and other information service activities / 7310 - Advertising
Digital Music Streaming & Downloads	Music Streaming & Download services offer unlimited access to content libraries for either monthly subscription fee or by purchasing them as per one-time transaction that afterwards allows permanent accessibility for the user.	SK: Personal, cultural, and recreational services	601: Radio broadcasting and audio distribution activities
Video on Demand	Video-on-Demand services can be 1) subscription-based – offering unlimited access to their content libraries for a monthly subscription fee; and 2) transaction based – offering time-limited access to video content that requires a usage-based one-time payment.	SK: Personal, cultural, and recreational services	6020: Television programming, broadcasting, and video distribution activities
eBooks	An eBook is the digital or electronic version of a book and can be read on various devices such as specific e-Readers as well as on tablets, smartphones, or computers.	SK: Personal, cultural, and recreational services	5811: Book publishing
Gaming Networks	Gaming Networks are paid subscription platforms for getting access to premium online video game content.	SI: Telecommunications, computer, and information services	5821: Publishing of video games
PC and Console Games	PC/console games are either video games that are sold online and which can be downloaded or played online.	SH: Charges for the use of intellectual property n.i.e. / SI: Telecommunications, computer, and information services	5821: Publishing of video games
Mobile Games	A mobile game is a video game designed to run on a mobile device such as a phone, tablet, or watch.	SH: Charges for the use of intellectual property n.i.e. / SI: Telecommunications, computer, and information services	5821: Publishing of video games
Online Travel Market	Online Travel Market is a specific online marketplace acting as intermediary for third-party vendors of short- and long-term homestays and travel experiences.	SJ: Other business services	5591: Intermediation services for accommodation<br>523: Intermediation services for transportation
Online Dating	Online Dating are digital services that offer a platform on which its members can flirt, chat or fall in love.	SK: Personal, cultural, and recreational services	9640: Intermediation services for other personal services
Online Education	Online Education is the transfer of knowledge or skills through online platforms. This includes the areas of online university education, online learning platforms, and professional certificates.	SK: Personal, cultural, and recreational services	8561: Intermediation services for courses and tutors, 85: Education

Supplementary Table 1 Continued. List of digital product sectors included in the dataset.

Digital product sector	Description	EBOPS correspondence	ISIC correspondence
Online Food Ordering	Online food ordering is the process of ordering food, for delivery or pickup, from a website or other application.	SJ: Other business services	5622: Intermediation services for food and beverage services activities
Online Gambling	Online gambling is any kind of gambling conducted on the internet. This includes virtual poker, casinos, and sports betting.	SJ: Other business services	9200: Gambling and betting activities
Online Marketplace, not elsewhere specified	An online marketplace, n.e.s., is a website acting as an intermediary for third-party vendors to offer their products and services to customers that is not included in Online Travel Market, Online Food Ordering, and Online Ride-Hailing.	SJ: Other business services	9640: Intermediation services for other personal services, 479: Intermediation services for retail trade, 6290: Other information technology and computer service activities
Online Ride-Hailing	Online Ride hailing services are online marketplaces that connect passengers and local drivers using their personal vehicles.	SJ: Other business services	523: Intermediation services for transportation
Operating System	An operating system is system software that manages computer hardware and software resources and provides common services for computer programs.	SH: Charges for the use of intellectual property n.i.e. / SI: Telecommunications, computer, and information services	5829: Other software publishing
Payment Service	A payment service is a system that enables digital financial transactions between merchants and customers through various channels such as credit cards or bank accounts.	SG: Financial services	6419: Other monetary intermediation
Business Intelligence Software	Business Intelligence Software is used for analyzing, visualizing, and presenting data and information in business context for rational business decisions. These tools help to access data, implement queries, create reports, and perform predictive analytics.	SI: Telecommunications, computer, and information services	5829: Other software publishing
Customer Relationship Management Software	Customer Relationship Management Software is designed to help companies to manage the entire life cycle of a customer including sales, marketing, customer services and contact center.	SH: Charges for the use of intellectual property n.i.e.	5829: Other software publishing
Enterprise Resource Planning Software	Enterprise Resource Planning Software is software that helps companies to manage, integrate and optimize important business activities related to their resources. These are oriented towards the company itself and its internal business processes.	SH: Charges for the use of intellectual property n.i.e.	5829: Other software publishing
Other Enterprise Software	Other Enterprise Software aggregates revenues for enterprise software that is not specifically mentioned in the other software sectors. This includes, for example, content applications, management software, performance management software etc.	SH: Charges for the use of intellectual property n.i.e.	5829: Other software publishing
Supply Chain Management Software	Supply Chain Management Software is software that supports supply- and demand side-processes within a company to offer a product or service on the market.	SH: Charges for the use of intellectual property n.i.e.	5829: Other software publishing
Administrative Software	This is software used to perform administrative tasks within businesses or organizations. It comprises software for the administration of IT infrastructure as well as standalone human resources and payroll management software.	SH: Charges for the use of intellectual property n.i.e.	5829: Other software publishing
Collaboration Software	Collaboration Software contains software that is designed to support collaboration within an organization such as conferencing and email applications as well as file synchronization and sharing applications.	SH: Charges for the use of intellectual property n.i.e.	5829: Other software publishing
Creative Software	Creative Software includes single purpose visualization, sound- and video recording, and editing software.	SH: Charges for the use of intellectual property n.i.e.	5829: Other software publishing
Office Software	Office Software is a collection of productivity software including at least a word-processor, spreadsheet, and a presentation program.	SH: Charges for the use of intellectual property n.i.e.f	5829: Other software publishing

Supplementary Table 2. List of Countries Included in the Digital Trade Dataset.

Country			
Afghanistan	Ecuador*	Lesotho	San Marino
Albania	Egypt*	Liberia	São Tomé and Príncipe
Algeria	El Salvador	Libya	Saudi Arabia*
Andorra	Equatorial Guinea	Lithuania	Senegal
Angola	Estonia	Luxembourg	Serbia
Antigua and Barbuda	Eswatini	Macau	Seychelles
Argentina*	Ethiopia	Madagascar	Sierra Leone
Armenia	Faroe Islands	Malawi	Singapore*
Australia*	Federated States of	Malaysia*	Slovakia
Austria*	Fiji	Maldives	Slovenia
Azerbaijan*	Finland*	Mali	Solomon Islands
Bahrain	France*	Malta	Somalia
Bangladesh	French Polynesia	Mauritania	South Africa*
Barbados	Gabon	Mauritius	South Korea*
Belarus*	Georgia	Mexico*	Spain*
Belgium*	Germany	Moldova	Sri Lanka
Belize	Ghana	Mongolia	Sudan
Benin	Greece*	Morocco	Suriname
Bermuda	Greenland	Mozambique	Sweden*
Bhutan	Grenada	Myanmar	Switzerland*
Bolivia	Guatemala	Namibia	Taiwan*
Bosnia and Herzegovina	Guinea	Nepal	Tajikistan
Botswana	Guinea-Bissau	Netherlands*	Tanzania
Brazil*	Guyana	New Caledonia	Thailand
Brunei	Haiti	New Zealand*	The Bahamas
Bulgaria	Honduras	Nicaragua	The Gambia
Burkina Faso	Hong Kong*	Niger	Togo
Burundi	Hungary*	Nigeria*	Tonga
Cambodia	Iceland	North Macedonia	Trinidad and Tobago
Cameroon	India*	Norway*	Tunisia
Canada*	Indonesia*	Oman	Turkey*
Cape Verde	Iran	Pakistan*	Turkmenistan
Cayman Islands**	Iraq	Panama	Tuvalu
Central African Republic	Ireland*	Papua New Guinea	Uganda
Chad	Israel*	Paraguay	Ukraine*
Chile*	Italy*	Peru*	United Arab Emirates*
China*	Ivory Coast	Philippines*	United Kingdom*
Colombia*	Jamaica	Poland*	United States*
Comoros	Japan*	Portugal*	Uruguay
Costa Rica*	Jordan	Qatar	Uzbekistan
Croatia	Kazakhstan*	Republic of the Congo	Vanuatu
Cuba	Kenya	Romania*	Venezuela
Cyprus*	Kiribati	Russia*	Vietnam*
Czechia*	Kuwait*	Rwanda	Yemen
Denmark*	Kyrgyzstan	Saint Kitts and Nevis	Zimbabwe*
Djibouti	Laos	Saint Lucia	
Dominica	Latvia	Saint Vincent and the	
Dominican Republic*	Lebanon*	Samoa	

Note: * Countries with available consumption data in the AppMagic dataset. ** Countries for which we only have export data (estimated using data on subsidiaries via optimal transport).

Supplementary Note 2. Digital revenue and consumption yearly summary statistics

In Supplementary Figure 1, we present the yearly summary statistics for the digital revenue and consumption data we have gathered.

Supplementary Figure 1 a depicts the total revenue generated from the digital products in our dataset over time. It is evident that the revenue has experienced exponential growth—from approximately USD 644 billion in 2016 to USD 1.85 trillion in 2021.

Supplementary Figure 1 b showcases the total consumption observed in AppMagic data (mobile applications and games) from 2016 to 2021. The total consumption for these digital sectors escalated from USD 30 billion in 2016 to over USD 80 billion in 2021.

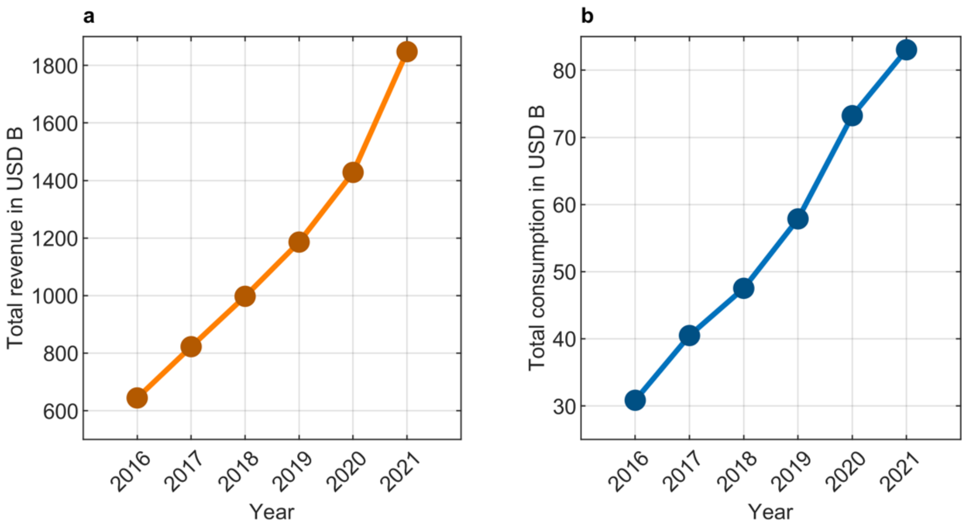

Supplementary Figure 1. Digital revenue and consumption summary statistics . **a** Total digital revenues of over time observed in our dataset . **b** Total consumption of mobile apps and games over time (from AppMagic)

In Supplementary Figure 2, we illustrate the distribution of revenues across digital sectors over the years. Within our dataset, the bulk of the revenue each year is consistently dominated by three specific sectors: cloud computing, digital advertising, and online marketplaces, n.e.s..

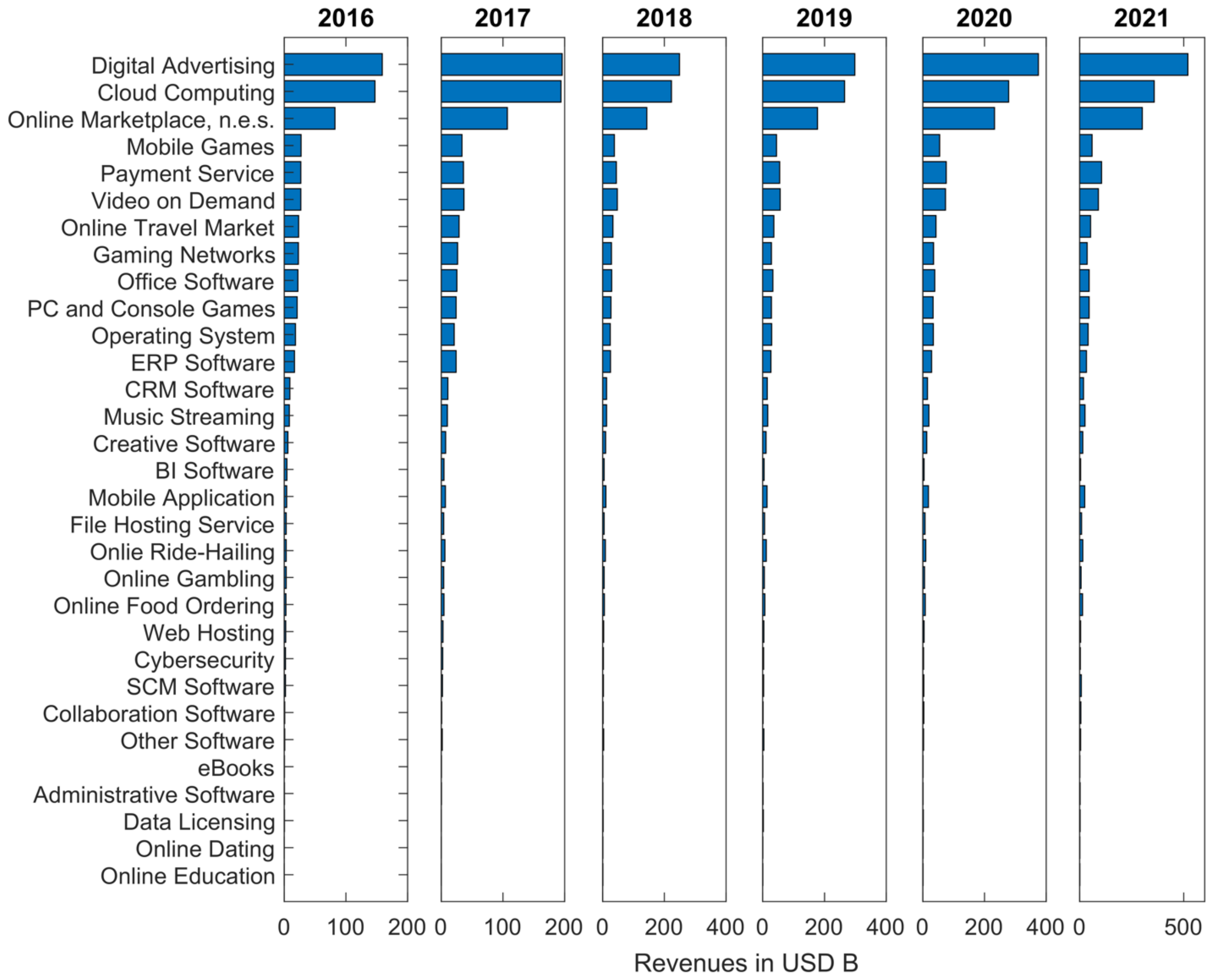

Supplementary Figure 2. Sectoral distribution of digital revenues over the years. Bar charts for the digital product consumption per sector for each year from 2016 until 2021. The digital product sectors are ordered according to their share in the world consumption in 2016.

Supplementary Note 3. Regression tree model features and cross-validation

3.1. Model features

Supplementary Table 3 lists the features used in our model and their data sources. We thereby emphasize that the digital revenue features are in their yearly value. Moreover, the economic size and ICT access features are four year averages (the values for 2020 are the average of the values between 2017-2020, and we exclude the missing values). This allows us generate feature values for certain countries that have missing data for a given year. Only the Geography and Cultural & historical features are fixed throughout the years. We also emphasize that in our regression tree model we always include logistic regression estimates for the probability of a non-zero consumption and dummy variables for the year of the observation (in the linear regression model this is equivalent to period fixed effects). The logistic regression uses the same features as the regression tree.

Supplementary Table 3. Features used to predict digital sector consumption across countries.

Feature	Data source (year)
**Digital revenue features**	
Total revenues of the firm in the digital sector (in USD) (in logs)	Own calculations using Orbis, Statista, and AppMagic data
Total digital revenues of all headquarters located in the country (in USD) (in logs)	Own calculations using Orbis, Statista, and AppMagic data
Total world revenues of the digital sector (in USD) (in logs)	Own calculations using Orbis, Statista, and AppMagic data
**Economic Size features**[*]	
GDP in current USD of the country of origin of the headquarters (in logs)	World Bank World Development Indicators
GDP in current USD of the country where the product is consumed (in logs)	World Bank World Development Indicators
**Geography features**	
Geographic distance (in km) between the most populated cities of the country of origin of the headquarters and the country where the product is consumed (in logs)[†]	CEPII Gravity database
World region of the country of origin of the headquarters	United Nations geoscheme
World region of the country where the product is consumed	United Nations geoscheme
Dummy variable for the contiguity between the country of origin of the headquarters and the country where the product is consumed	CEPII Gravity database
**Cultural & historical features**	
Dummy variable for common official language between the country of origin of the headquarters and the country where the product is consumed	CEPII Gravity database
Dummy variable for common unofficial language (spoken by at least 9% of the population) between the country of origin of the headquarters and the country where the product is consumed	CEPII Gravity database
Dummy variable describing whether the country of origin of the headquarters and the country where the product is consumed were ever in a colonial relationship	CEPII Gravity database
Dummy variable describing whether the country of origin of the headquarters and the country where the product is consumed shared a common colonizer post 1945.	CEPII Gravity database
Dummy variable describing whether the country of origin of the headquarters and the country where the product is consumed are currently in a colonial relationship	CEPII Gravity database
Dummy variable describing whether the country of origin of the headquarters and the country where the product is consumed were in colonial relationship post 1945	CEPII Gravity database
Dummy variable describing whether the country of origin of the headquarters and the country where the product is consumed were ever part of the same country	CEPII Gravity database
**ICT Access Features**	
Internet users as a share of population for the country of origin of the headquarters (in logs)	The International Telecommunication Union
Internet users as a share of population for the country where the product is consumed (in logs)	The International Telecommunication Union
Fixed broadband connections as a share of population for the country of origin of the headquarters (in logs)	The International Telecommunication Union
Fixed broadband connections as a share of population for the country where the product is consumed (in logs)	The International Telecommunication Union
Mobile broadband connections as a share of population for the country of origin of the headquarters (in logs)	The International Telecommunication Union
Mobile broadband connections as a share of population for the country where the product is consumed (in logs)	The International Telecommunication Union

[*] GDP data for Chinese Taipei and Venezuela come from IMF's World Economic Outlook.
[†] We set the distance to 0 when the country of origin is the same as the destination country.

3.2.Model cross-validation

We train our model by filtering out firm-digital sector pairs with revenues under USD 10 million and by using a group-K-fold cross-validation, where we leave 20% of the firm-category pairs as a test set, to tune the hyperparameters. The idea behind this cross-validation approach is that our main goal is to extend the consumption data to new digital categories.

We use a grid search over several possible values for maximum tree depth (1, 3, 5, 7) and minimum child weight (5, 20, 50, 100, 200), and fix the learn rate to 0.1 and the number of learning cycles to 150.

Supplementary Figure 3 provides a heatmap for the average cross-validated MSE across all hyperparameter choices. The minimum error occurs when the maximum tree depth is 3 and the minimum child weight is 100 (MSE = 23.14). For comparison, a linear regression model has an average MSE = 24.44. Hence, our model improves upon this baseline.

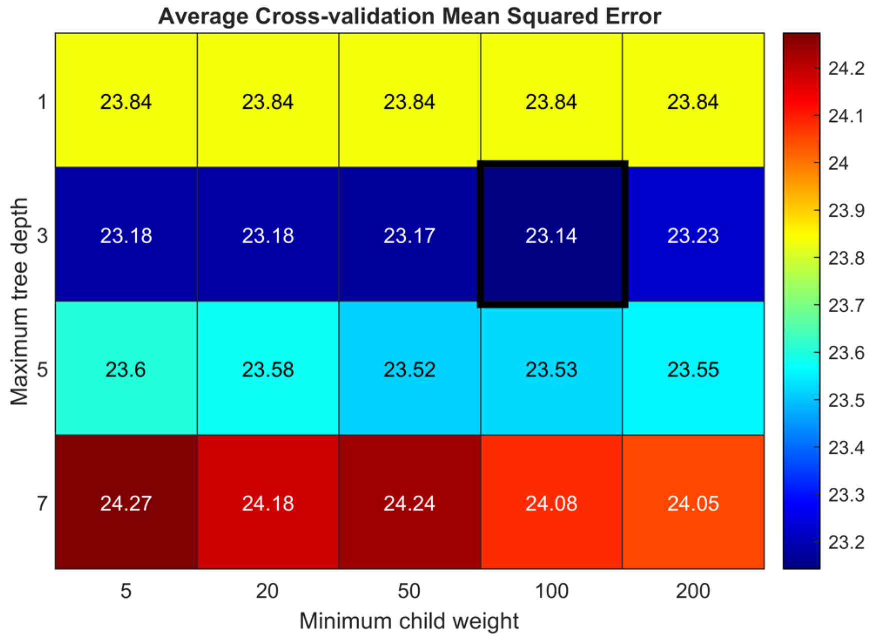

Supplementary Figure 3. Heatmap for the grid search average MSE. The best model (with lowest mean-squared error) is highlighted with a black box.

We also explore the model's performance in predicting the realized regional consumption share in 2021 for the parent firms with available data. Namely, from the annual reports of several firms included in our dataset, we were able to extract the regional consumption share (multiple parent companies report geographical revenue data for regions based on the location of the customers). Supplementary Figure 5 plots the scatter chat for the results from our cross-validated model and the results from the linear regression. We again observe that our model performs much better at predicting these shares (MSE = 0.048), compared to the linear regression (Pearson correlation = 0.126).

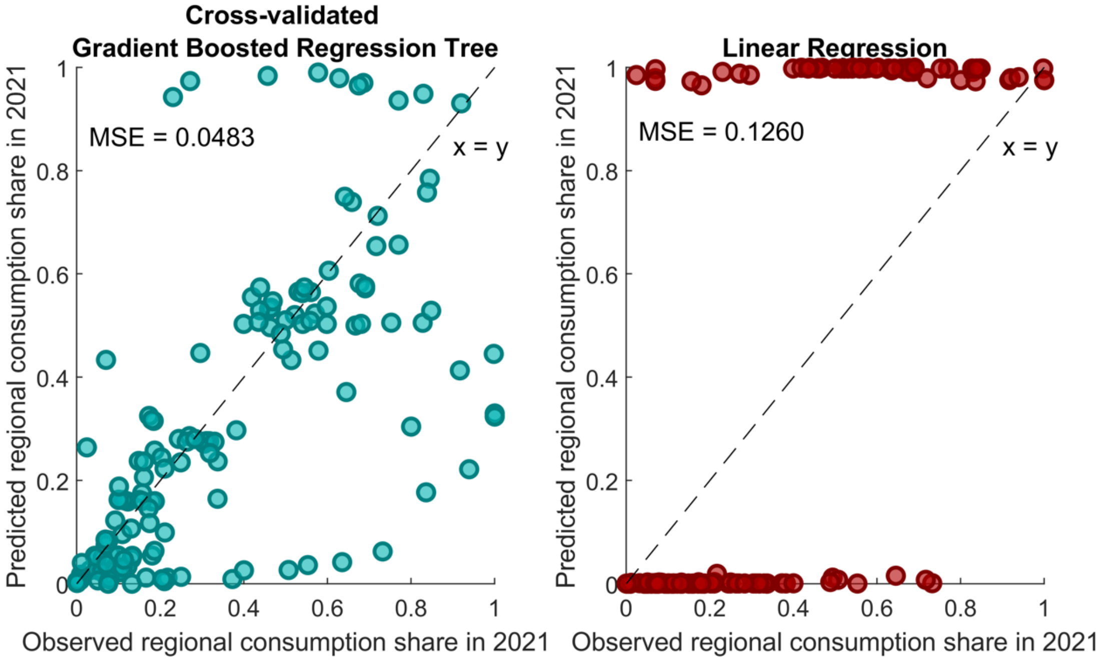

Supplementary Figure 4. Observed vs. predicted regional consumption shares in 2021 for the firms with available data. Scatter charts for the observed regional consumption share per for firms with available data against the predicted values from the gradient boosted regression tree (left panel) and the predicted values from the linear regression (right panel). The dashed diagonal line in both panels is the x=y line.

Supplementary Note 4. Results with alternate allocation procedures for digital products consumption

We also provide alternate results where we assign all product revenues to the locations of the headquarters of the firm-category pair. This alternate procedure should increase the exports of the headquarters' origin countries towards the world. It could also decrease the imports of these countries coming from the subsidiaries' origin countries if the headquarters use these countries to outsource the production processes.

Supplementary Figure 5 compares the volume of digital products trade over the years between our estimates based on subsidiary allocation (with optimal transport) and the alternate headquarters estimates (without optimal transport). For 2021, we find digital products trade to be slightly lower in volume when the optimal transport procedure is removed from the estimation, albeit this decrease is only marginal (4%). In the period between 2016 and 2019, digital products trade is only slightly higher if we opt for the headquarters assignment (between 4% and 6% higher). Hence, the global trends for digital products trade remain similar even when we allocate all the revenue to the headquarters.

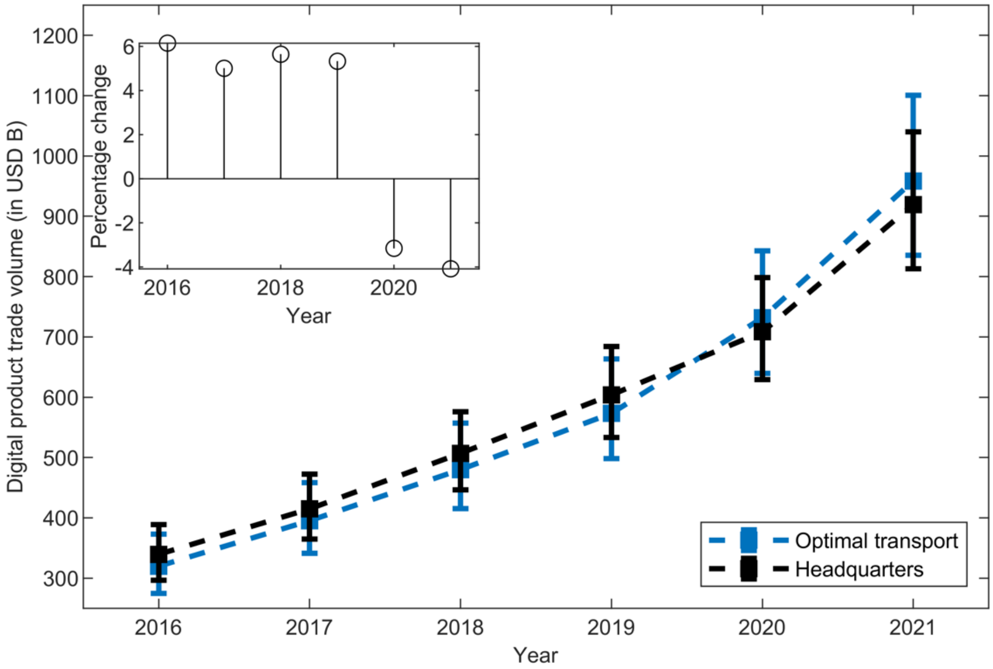

Supplementary Figure 5. Digital products trade over the years for our estimates using subsidiary assignment (with optimal transport) and the headquarters estimates (without optimal transport). The inset plot shows the percentage change from our baseline to the alternate estimates. The error bars show 95% confidence intervals.

Supplementary Figure 6 shows the geographical concentration of digital trade in the headquarters assignment, and compares it with the optimal transport results. In this case, digital products exports are even more concentrated (2 countries account for more than 80% of the exports). Imports have similar concentration.

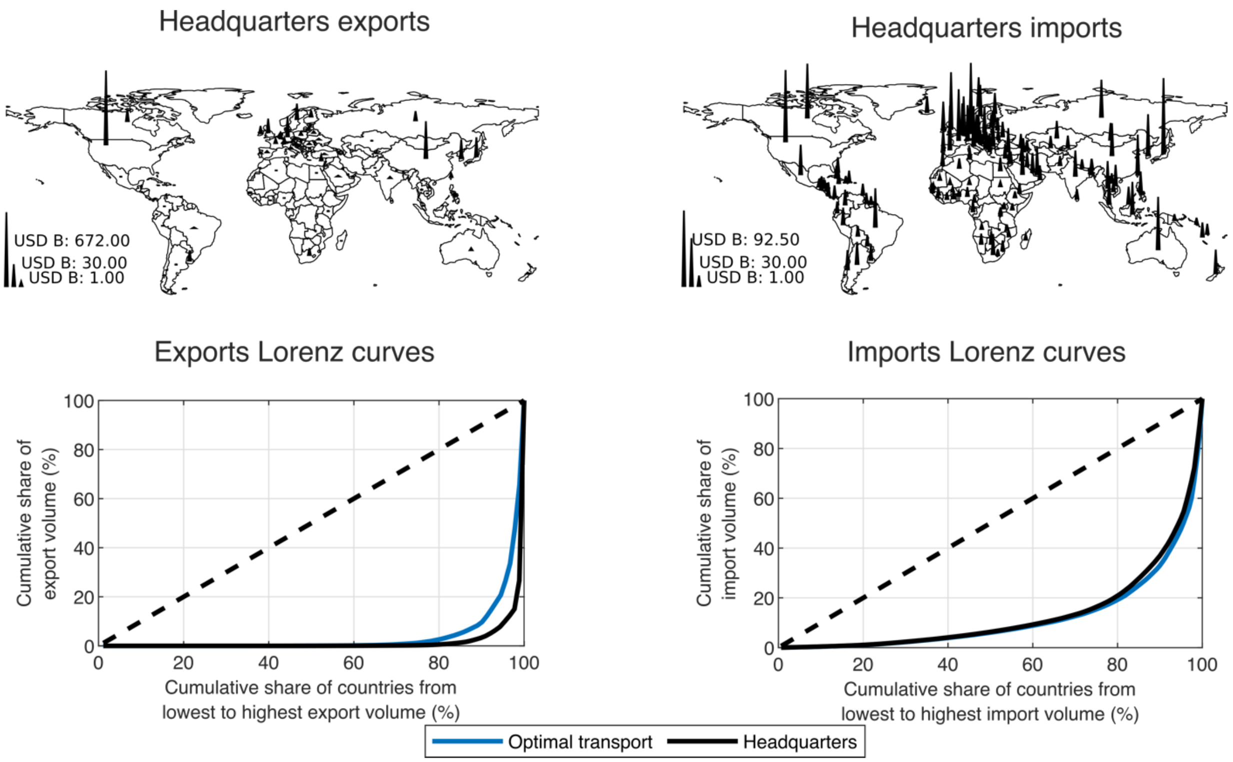

Supplementary Figure 6. The geography of digital products trade in 2021 using the headquarters assignment. Top panels provide spike maps for the distribution of digital product exports (top left panel) and digital product imports (top right panel) in 2021 using the headquarters assignment. Bottom panels provide Lorenz curves for the digital product exports (bottom left panel) and digital product imports (bottom right panel) in 2021 using the headquarters assignment.

Finally, Supplementary Figure 7 displays the network of digital product trade for the headquarters assignment. In this case, the centrality of the USA increases, and China becomes one of the most important exporters.

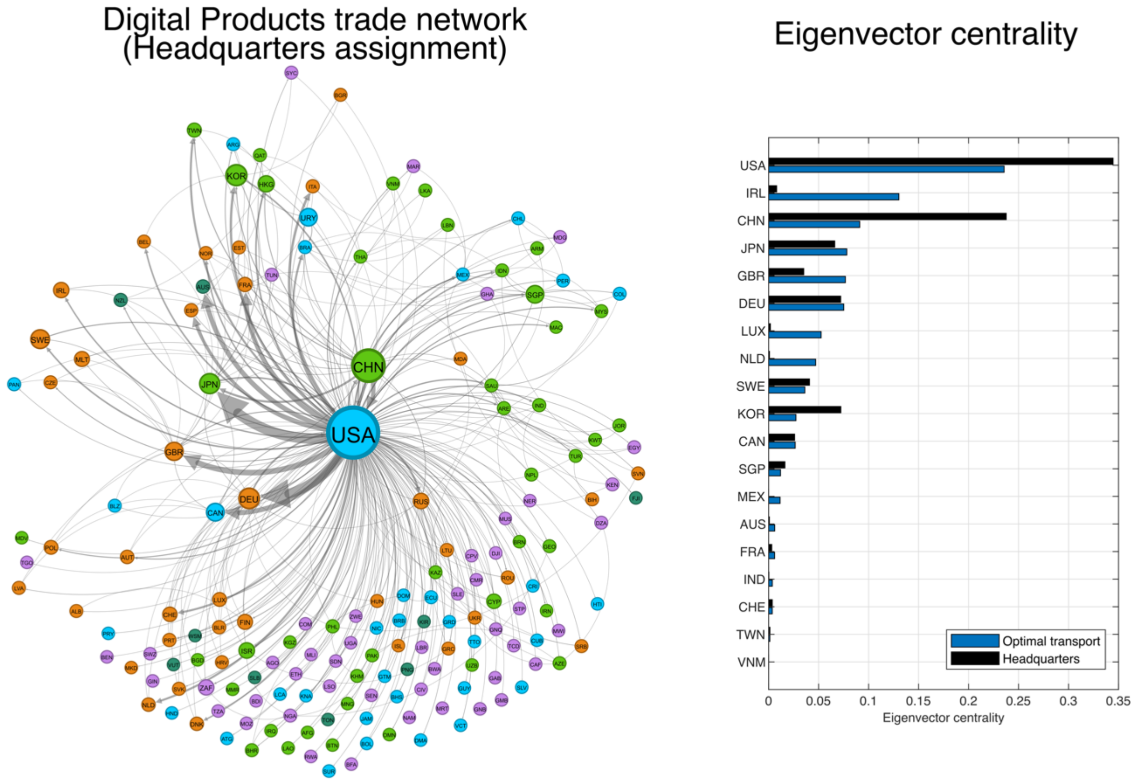

Supplementary Figure 7. The digital products trade network in 2021 with headquarters assignment. In the left panel, we reproduce the network visualization depicted in Figure 5 a from the main manuscript using the headquarters assignment. For each country we show the top import and export destination. We also highlight all bilateral trade flows with a volume above USD 1B. In the right panel, we show the Eigenvector centralities for the top 10 countries in terms of eigenvector centralities of the network in the left panel.

Supplementary Note 5. Structure of digital products trade over the years

In Supplementary Figure 8 we visualize the sectoral distribution of digital products trade between 2016 and 2021. We find that most of the trade in digital products between 2016 and 2021 is explained by Cloud Computing, Digital Advertising, and Online marketplaces, n.e.s..

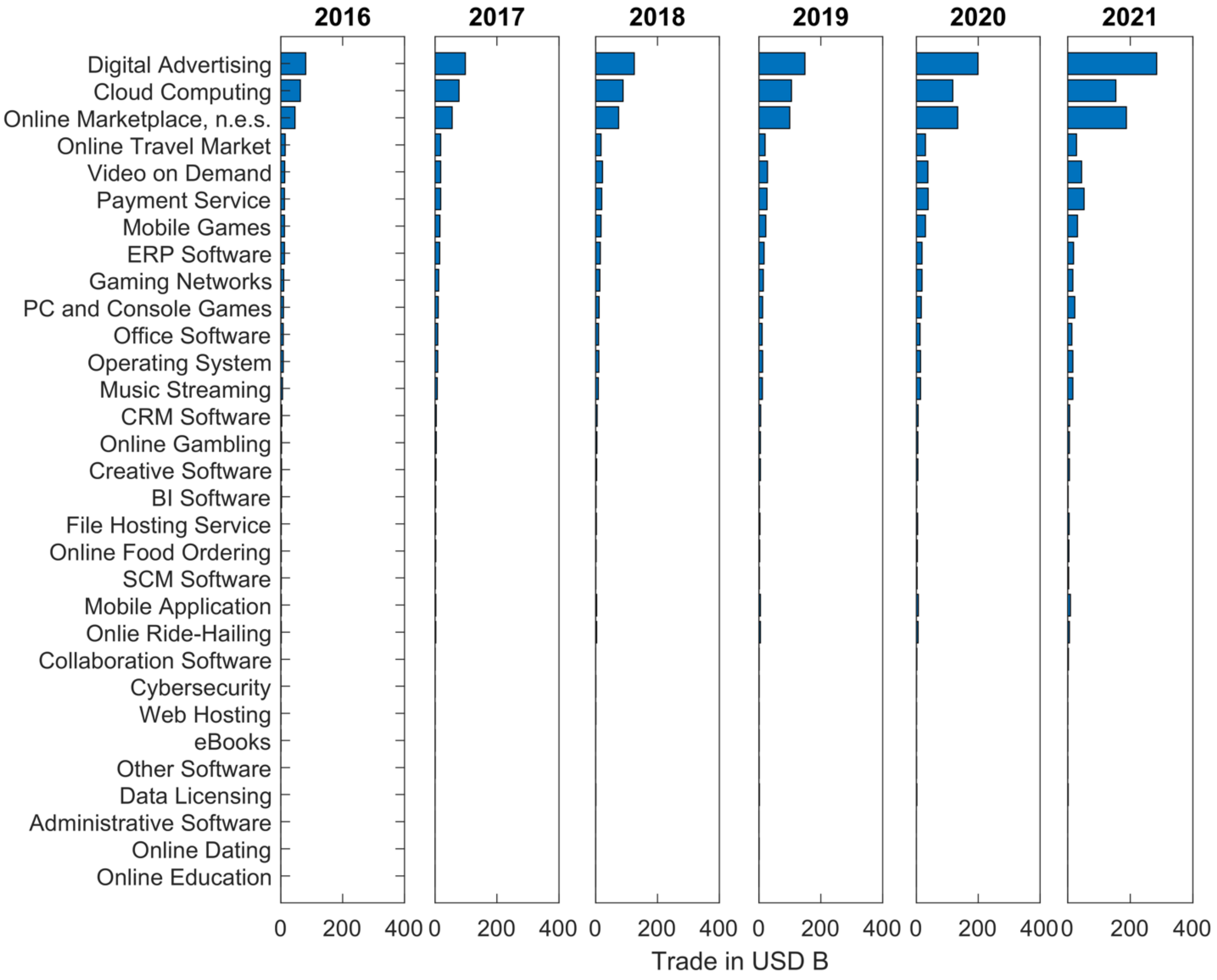

Supplementary Figure 8. Sectoral distribution of digital products trade over the years. Bar charts for the digital product trade per sector for each year from 2016 until 2021. The digital product sectors are ordered according to their share in the world trade in 2016.

Supplementary Note 6. Concentration of digital products trade

Here we compare in more detail the concentration of digital products exports, physical exports and services exports using the Shannon Entropy measure of concentration. The Shannon Entropy of a product category is defined as:

$$E_p = -\sum_c y_{cp} \log(y_{cp}),$$

where $y_c = \frac{X_{cp}}{\sum_c X_{cp}}$, with X_{cp} being the exports of country c in p, is the market share of the country in that product. Higher entropy values imply lower concentration.

We compare the Shannon Entropy of digital products trade to the Shannon Entropy of each of the 21 sections of the HS physical goods classification (the section definitions can be seen here: https://oec.world/en/product-landing/hs), and each of the 12 EBOPS 2010 categories (the definitions can be found here: https://unstats.un.org/unsd/classifications/Family/Detail/101). Also, we undertake a simulation where we randomly select HS4 products such that their total trade matches that of digital products trade. We estimate the Entropy of this random sample of products and use it as a benchmark to understand the unique concentration patterns of digital products trade (we repeat this process 1000 times and report the average Entropy).

Supplementary Figure 9 gives the results. We observe that digital products exports are more concentrated than all HS Section exports and EBOPS service categories exports. Also, digital exports are much more concentrated than our random baseline. These results strengthen the findings presented in the main manuscript about the underscoring the distinct dynamics that govern digital products trade.

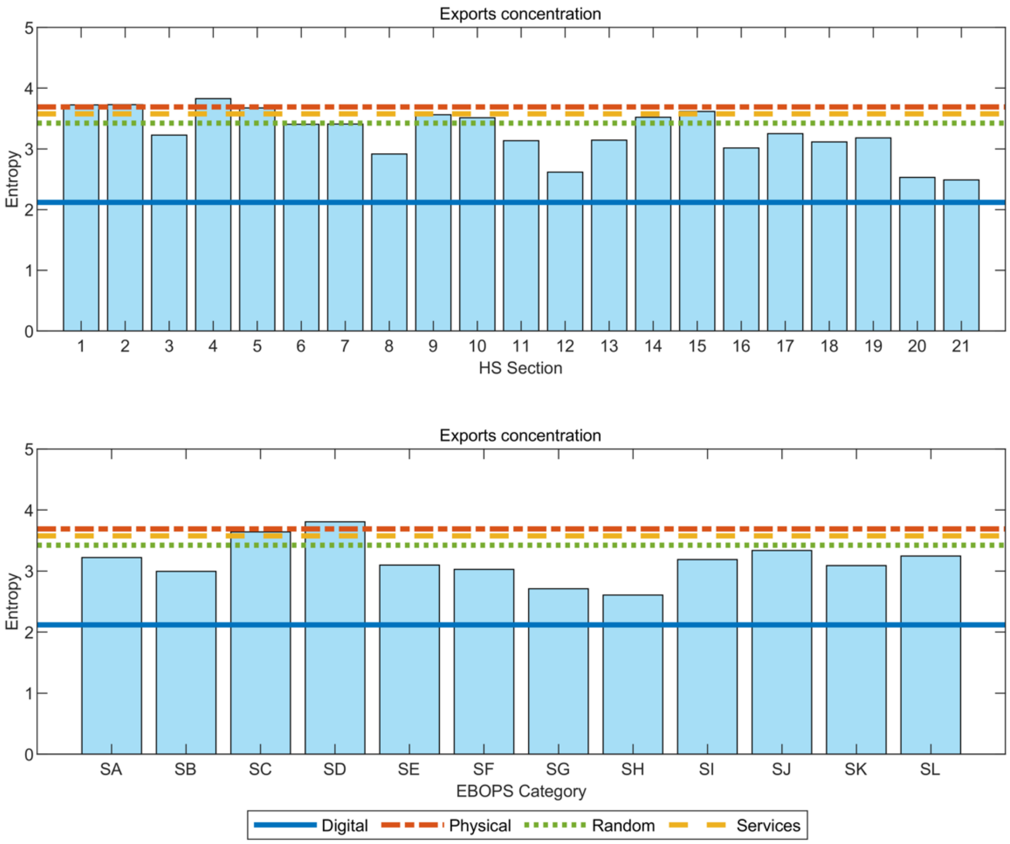

Supplementary Figure 9. Bar charts for the estimated Entropy (concentration) of physical exports and services exports compared to digital products exports. The top panel compares physical goods exports with digital product exports, whereas the bottom panel compares services exports with digital product exports.

Supplementary Note 7. Decoupling definitions and robustness checks

7.1.Decoupling definition

We formally define decoupling using the *Decoupling Index* (DI)[2–6]. For each country, this index can be calculated using the following equation

$$DI = \frac{\Delta\text{GDP\%} - \Delta\text{Em\%}}{\Delta\text{GDP\%}},$$

where,

$$\Delta\text{GDP\%} = \frac{GDP_1 - GDP_0}{GDP_0},$$

is the relative change in GDP per capita between 2016 and 2019, (GDP_1 is the GDP per capita in 2019 and GDP_o is the GDP per capita in 2016), and

$$\Delta\text{Em\%} = \frac{Em_1 - Em_0}{Em_0},$$

is the relative change in greenhouse gas emissions in kilotons CO2 per capita between 2016 and 2019 (Em_1 is the greenhouse gas emissions in kilotons CO2 per capita in 2019 and Em_o is the greenhouse gas emissions in kilotons CO2 in 2016).

A country that has decoupled economic growth from emissions has a DI of above 1. This is also known as “absolute decoupling” and refers to a decline of emissions in absolute terms while GDP per capita grows.

For this analysis, we collect the data for GDP per capita (in PPP 2017 USD) and population from the World Bank development indicators. Production and consumption emissions are from the Global Carbon Budget[7]. In the analysis, we include only countries that had a population of above

1.5 million in 2021. Also, countries that had negative economic growth during the period of analysis are excluded.

7.2. Decoupling between emissions and growth for all countries

We investigate the robustness of our results given in Figure 6 c from the main text, by re-estimating the trade patterns using data on all countries.

Supplementary Figure 10 gives the results. We again observe that the 25th percentile of the exports per capita for the countries that have achieved decoupling are similar in volume to the median of the non-decoupling economies (See also Supplementary Figure 11 where we zoom in on the relationship between the median for the decoupled and the 25th percentile for the non-decoupled). In this case, though, the 25th percentile of the exports per capita of DDS, services, and goods for the countries that have achieved decoupling are also similar to the median of the non-decoupling economies.

Since a large group of economies do not have digital product exports, we also provide a more conservative estimate using data on all countries that with non-zero digital products exports in a year in our dataset. Supplementary Figure 12 gives the results for all types of trade (digital products, DDS, services, and goods), whereas Supplementary Figure 13 zooms in on the relationship between the median for the decoupled and the 25th percentile for the non-decoupled for this sample. We again find that the : 25th percentile of the exports per capita of digital products, is similar to the median of the non-decoupling economies.

We point out that these results for the sample including all countries might be driven by the variance in development levels. This is particularly relevant given that high-income economies are

more likely to have both the capacity for significant exports and the resources to invest in decoupling efforts. Consequently, by including economies at all income levels, we potentially introduce a bias whereby the export capabilities of decoupled, typically wealthier economies are overstated when compared to their less developed counterparts.

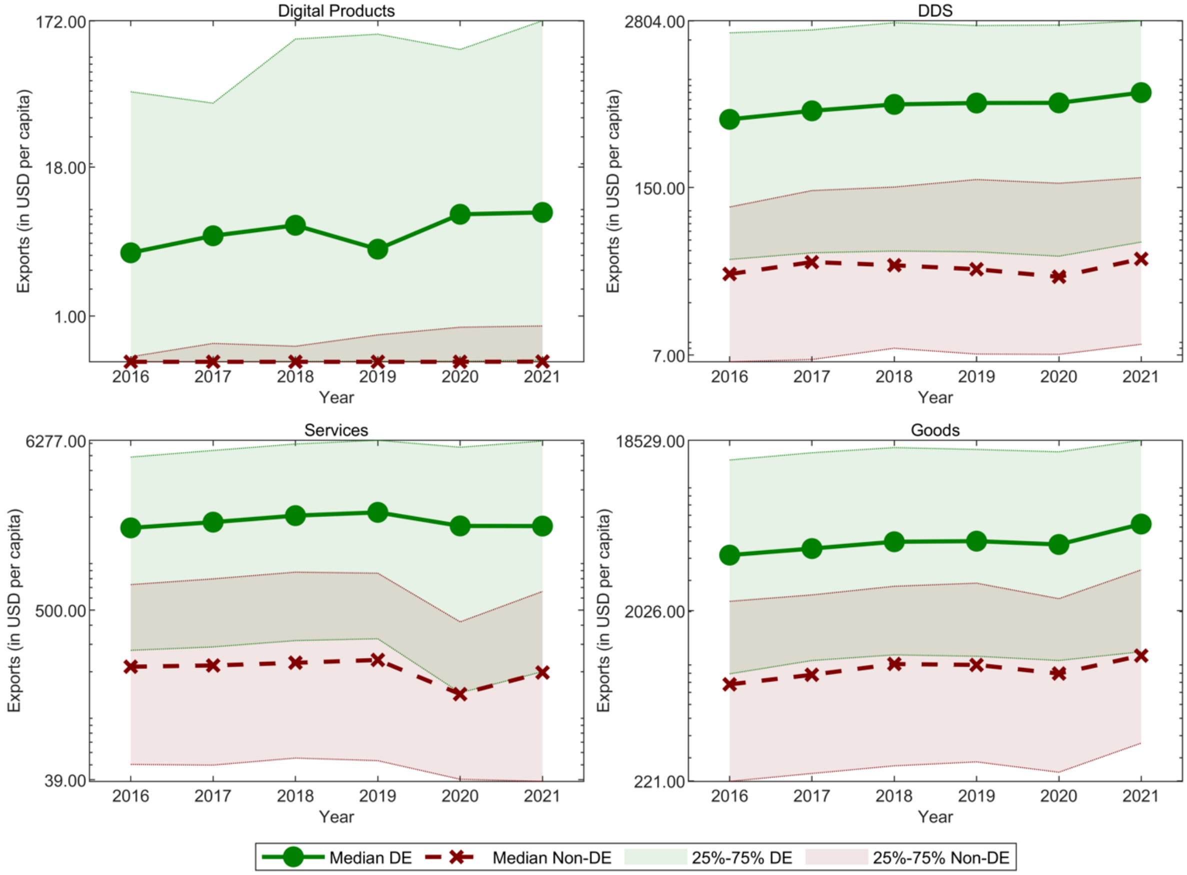

Supplementary Figure 10. Digital products trade and twin transition based on production emissions and using data on all economies. Median digital, DDS, services, and goods exports per capita between 2016 and 2021 for all economies depending on whether they decoupled growth from emissions or not. In the legend: DE – decoupled, non-DE – non-decoupled.

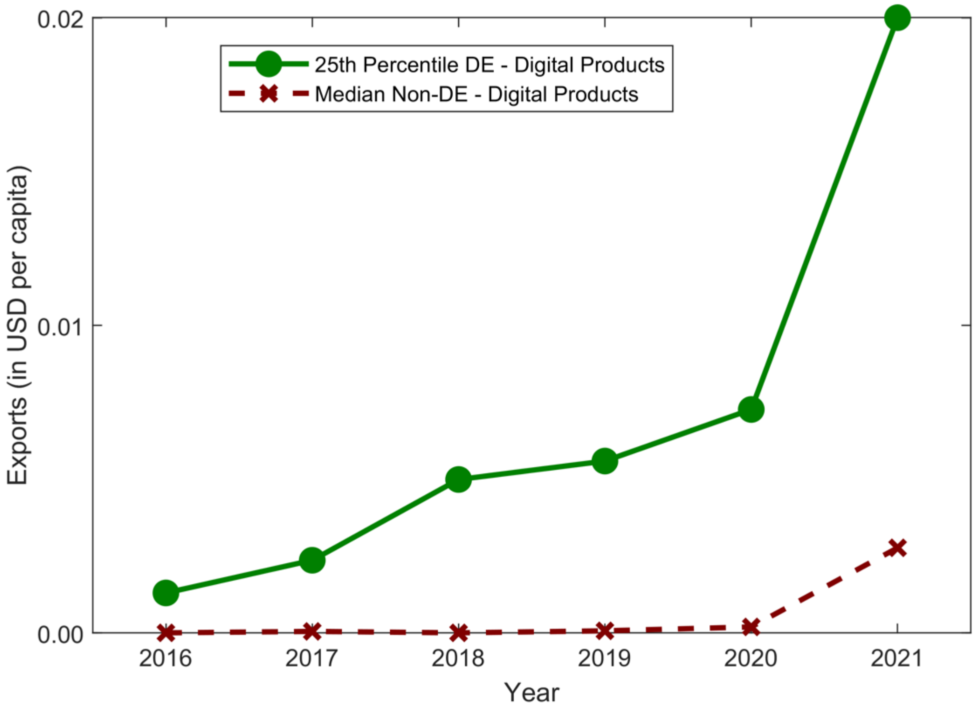

Supplementary Figure 11. 25th percentile digital exports per capita between 2016 and 2021 for all economies that have decoupled growth from emissions and the median for those that have not decoupled emissions from growth. In the legend: DE – decoupled, non-DE – non-decoupled.

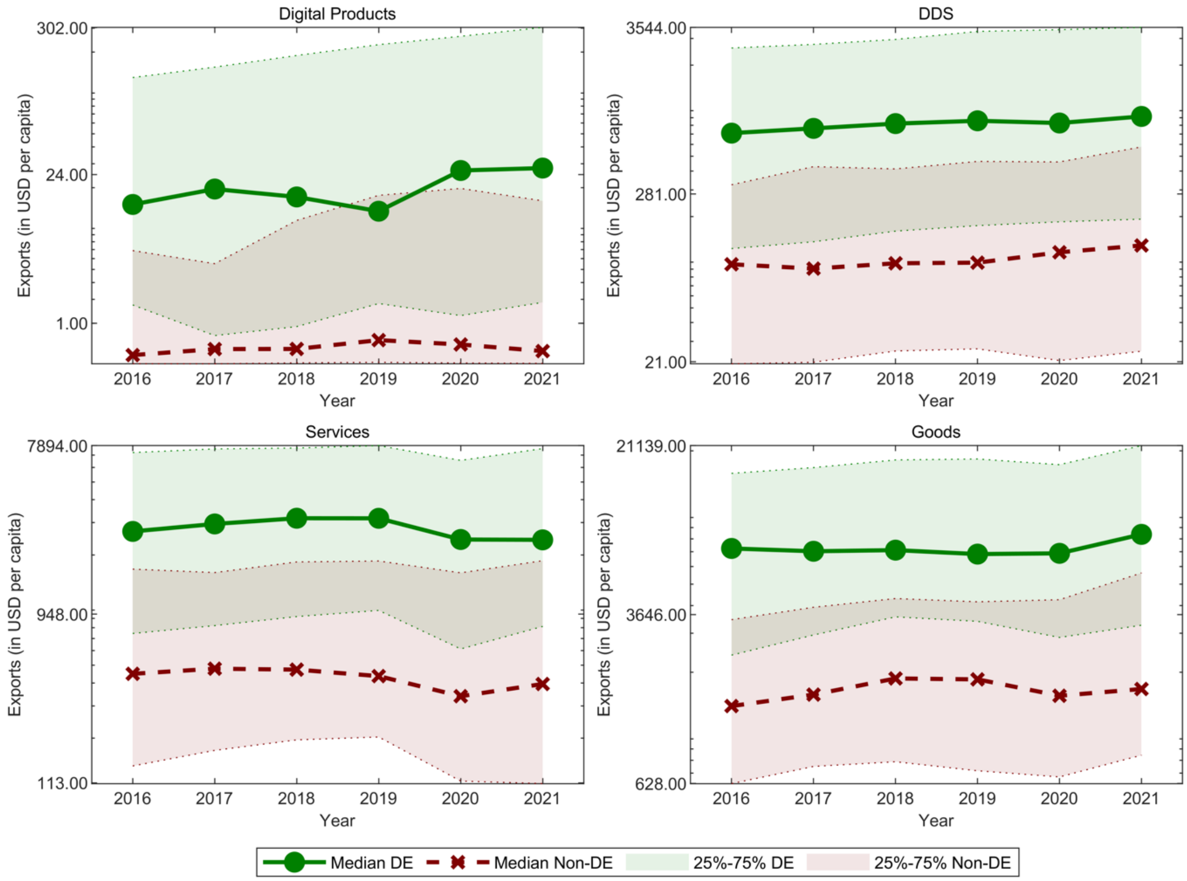

Supplementary Figure 12. Digital products trade and twin transition based on production emissions and using data on all economies with non-zero digital products exports. Median digital, DDS, services, and goods exports per capita between 2016 and 2021 for all economies with non-zero digital products exports depending on whether they decoupled growth from emissions or not. In the legend: DE – decoupled, non-DE – non-decoupled.

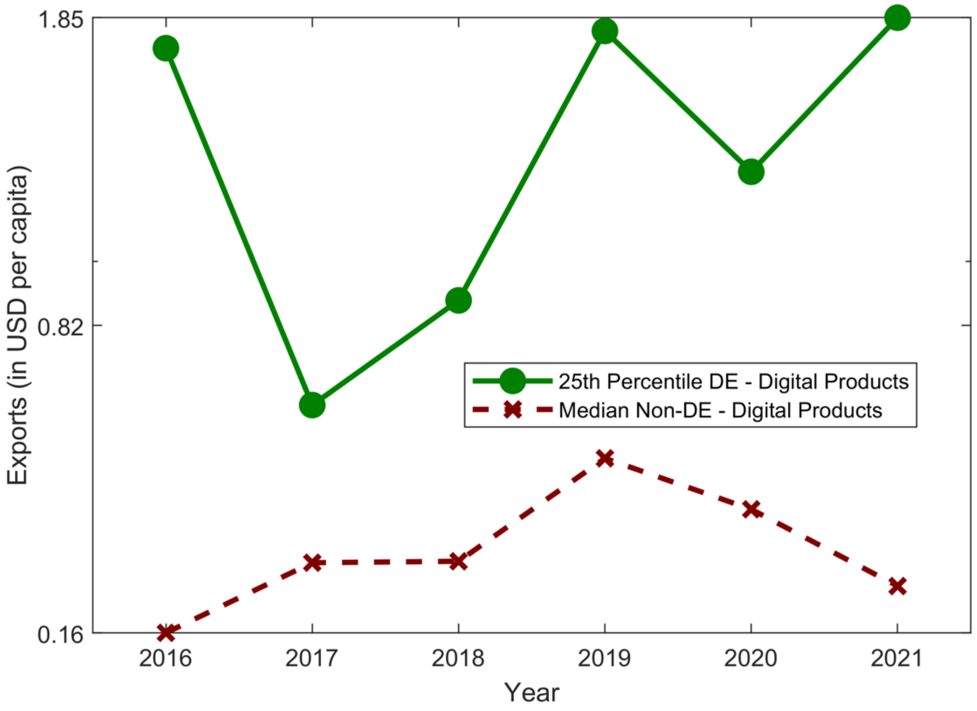

Supplementary Figure 13. 25th percentile digital exports per capita between 2016 and 2021 for all economies with non-zero digital products exports and that have decoupled growth from emissions, and the median for those that have not decoupled emissions from growth. In the legend: DE – decoupled, non-DE – non-decoupled.

7.3. Decoupling between emissions and growth for consumption emissions

As a second robustness check, we re-estimate the decoupling on the basis of consumption emissions.

Supplementary Figure 14 gives the results for the restricted sample of high income economies, whereas. Again, we observe that the 25th percentile of the per capita digital product exports for the decoupled economies is slightly higher than the median for the non-decoupling economies. In this case, the 25th percentile of the DDS, services, and goods exports for the decoupled economies is not higher than the median of the non-decoupled economies, but it is still of similar size.

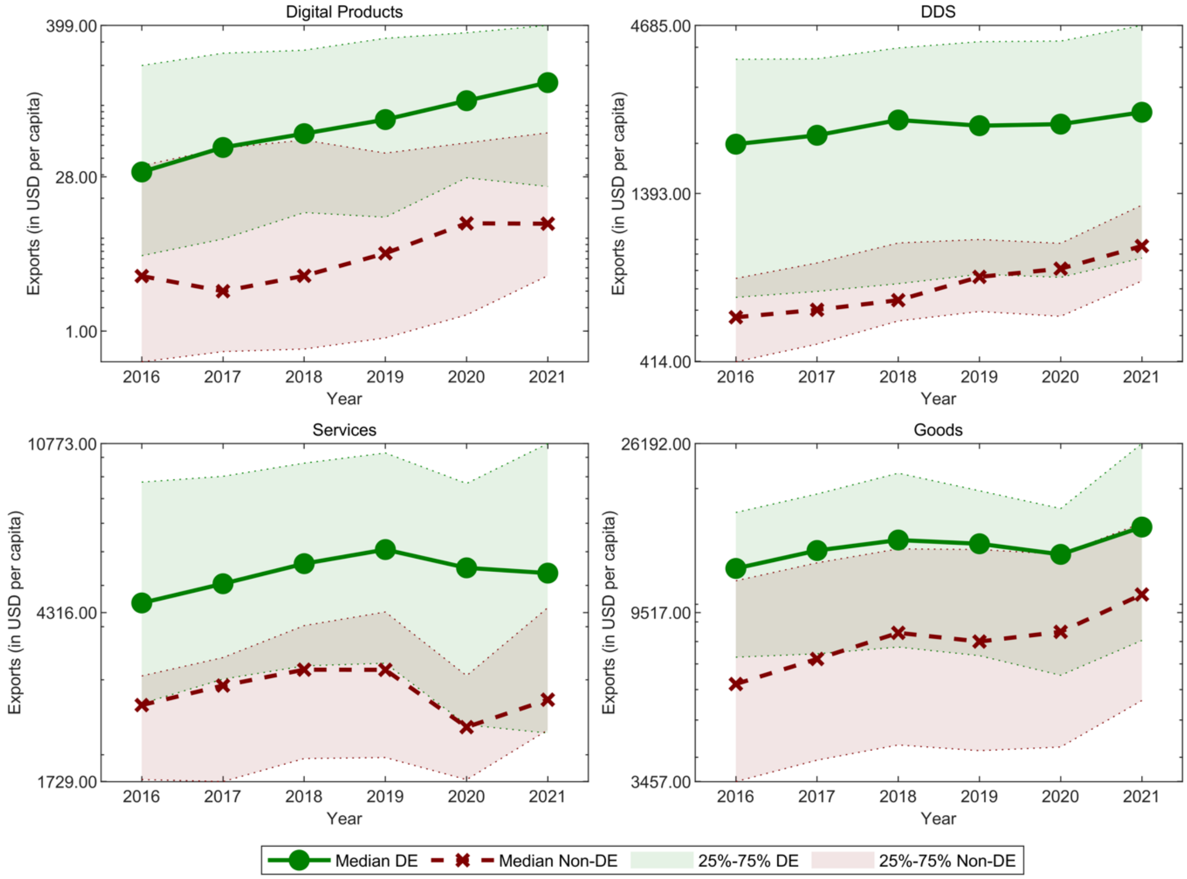

Supplementary Figure 14. Digital products trade and twin transition based on consumption emissions (only high income economies). Median digital exports per capita between 2016 and 2021 for high income economies that have decoupled growth from emissions and the 75th percentile for those that have not decoupled emissions from growth. Decoupling is estimated using consumption emissions. In the legend: DE – decoupled, non-DE – non-decoupled.

Supplementary Note 8. Economic complexity definitions, rankings, and regression analyses

8.1. Economic Complexity Index definition

Economic complexity metrics are derived from specialization matrices, summarizing the geography of multiple economic activities (using dimensionality reduction techniques akin to Singular Value Decomposition or Principal Component Analysis) [8,9]. In particular, given an output matrix X_{cp}, summarizing the exports, patents, or publications of an economy *c* in an activity *p*, we can estimate the economic complexity index ECI_c of an economy and the product complexity index PCI_p of an activity, by first normalizing and binarizing this matrix:

$$R_{cp} = {}^{X_{cp}X}\!\Big/_{X_p X_c},$$
$$M_{cp} = \begin{cases} 1 & if\ R_{cp} \geq 1 \\ 0 & otherwise \end{cases}, \tag{1}$$

where muted indexes have been added over (e.g., $X_p = \sum_c X_{cp}$) and R_{cp} stands for the revealed comparative advantage of economy *c* in activity *p*. Then, we define the iterative mapping:

$$ECI_c = \frac{1}{M_c} \sum_p M_{cp} PCI_p,$$
$$PCI_p = \frac{1}{M_p} \sum_c M_{cp} ECI_c\ . \tag{2}$$

That is, according to (2), the complexity of an economy *c* is defined as the average complexity of the activities *p* present in it (and vice-versa). The normalization steps in (1) and (2) are required to make the units of observation comparable (e.g. China and Uruguay are very different in terms of size). The solution of (2) can be obtained by calculating the eigenvector corresponding to the second largest eigenvalue of the matrix:

$$M_{cc'} = \sum_{pc'} \frac{M_{cp} M_{c'p}}{M_c M_p} \tag{3}$$

Which is a matrix of similarity between economies c and c' normalized by the sum of the rows and columns of the binary specialization matrix M_{cp} (it considers similarity among economies counting more strongly rare coincidences).

To obtain ECI_c, the values of the eigenvector are normalized using a z-score transformation (meaning that the average complexity is 0).

We build our results using the standard definition of ECI[8,10] because this is a widely used definition, making our results more readily comparable with previous research.

8.2. Digital product complexity rankings

Supplementary Figure 15 gives the PCIs of all digital products in 2021. The most complex digital products were Digital Advertising, eBooks, and File Hosting Services whereas the least complex was Online Food Ordering.

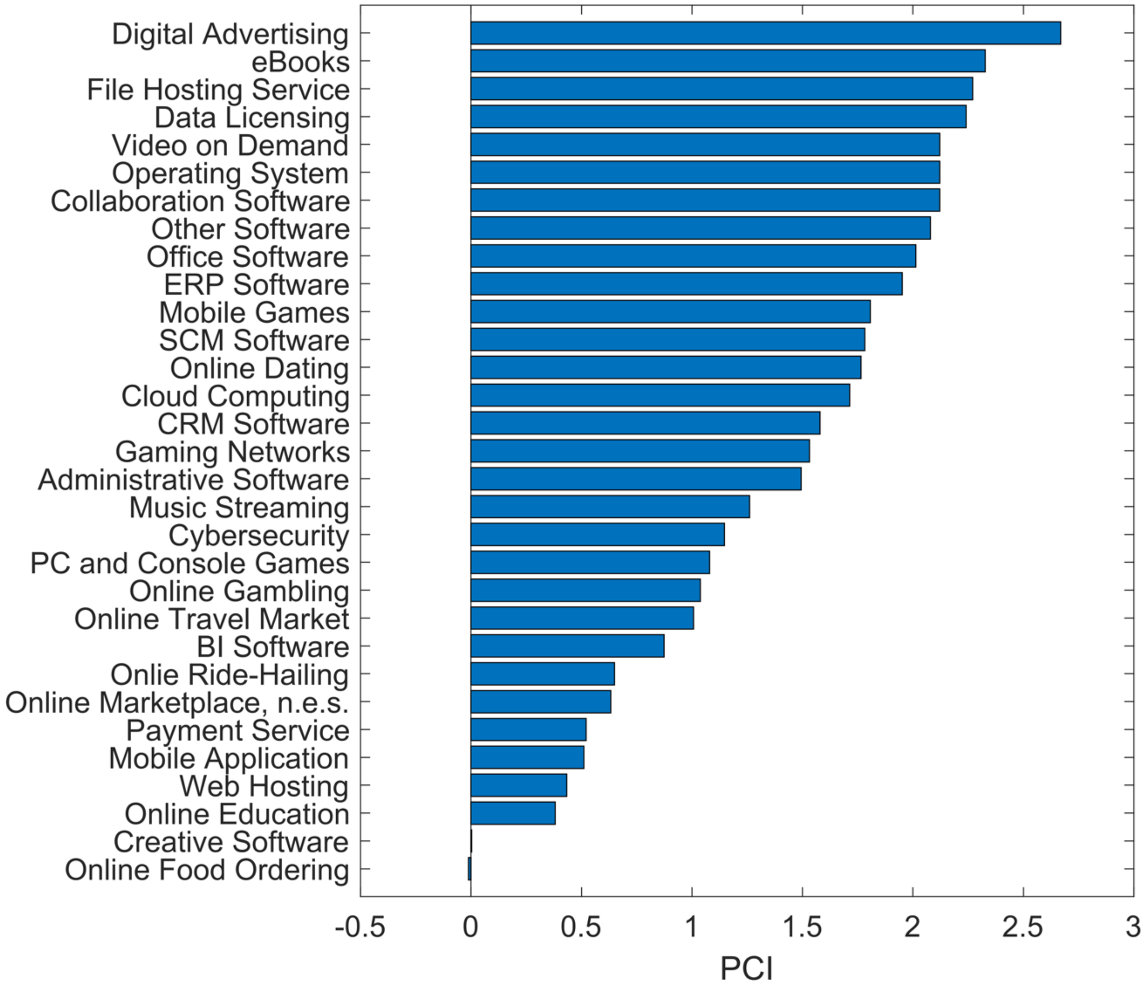

Supplementary Figure 15. Digital products complexity rankings in 2021. Bar chart for the digital products complexity rankings in 2021. The digital product sectors are ordered according to their complexity.

8.3. Complexity in digital products and services and inclusive green growth

We also explore how complexity in digital product and services could contribute to explaining inclusive green growth by following the economic complexity literature[10–12]. Specifically, for this analysis we follow the setup of Stojkoski et al.[13] and explore whether the combined ECI including physical and digital products trade could improve the explanatory power of the model using just physical trade in explaining international variations in economic growth. That is, we set up cross-country regressions of the form

$$y_c = b\, ECI_c + a^T X_c + b_0 + e_c,$$

where y_c is the dependent variable for country c (economic growth, emission intensity), ECI_c is the economic complexity index of the country (estimated with just physical trade, or by combining physical and digital products trade), X_c is a vector of control variables that account for other key factors (e.g. population, GDP per capita, etc.), b_0 is the intercept, and e_c is the error term. We refer to Stojkoski et al.[13] for formal definitions and data sources of the variables used in our regression models.

Note that, the ECI has also often been related with income inequality (Gini index). However, due to limited time-series (the data used in Stojkoski et al.[13] for exploring the income inequality relationship is limited to 2015), we are unable to explore this relationship. Also, the regression analyses in Stojkoski et al.[13] are using panel data. Again, due to limited time-series here we restrict ourselves to the latest period.

Economic growth: We test the effect of digital products trade on economic growth by looking at the annualized GDP per capita growth (in constant 2017 PPP dollars) between 2016 and 2021 (the longest period with available data). The baseline model includes the log of the initial GDP per capita (in constant 2017 PPP dollars). This captures Solow's idea of economic convergence[14] (baseline model is presented in column 1 of Supplementary Table 4).

Supplementary Table 4 shows the effect of digital products trade on the ability of economic complexity metrics to explain economic growth. We find that physical trade complexity (ECI (physical)) is a significant and positive predictor of economic growth (column 2). Moreover, ECI (physical) is robust when adding other potential covariates: natural resources, goods exports per capita, and population (column 3, see Stojkoski et al.[13] for formal definitions and data sources).

Combining physical trade and digital products trade (ECI (physical and digital)) yields similar results (columns 4 and 5 of Supplementary Table 4). When we include ECI (physical and digital) and ECI (physical) in one regression model together, we find that they are not robust together (column 6 of Supplementary Table 4), i.e., they change sign. Hence, both complexity indexes have similar power in explaining economic growth. Nevertheless, we emphasize that our models are restricted to the latest 6 years, and as such, have limited explanatory power. We believe that with

further advancements in data availability and the inclusion of longer time-series, we can build upon this preliminary analysis to construct even more nuanced models.

Supplementary Table 4. Economic Growth Regression Results.

	Dependent variable:					
	GDP per capita growth (2016-2021)					
	(1)	(2)	(3)	(4)	(5)	(6)
ECI (digital and physical)				1.373***	1.180**	3.502
				(0.410)	(0.567)	(6.962)
ECI (physical)		1.353***	1.152**			-2.307
		(0.404)	(0.561)			(6.874)
Log of exports per capita			-0.169		-0.182	-0.183
			(0.948)		(0.944)	(0.946)
Log of natural resource exports per capita			-0.496		-0.492	-0.506
			(0.819)		(0.814)	(0.820)
Log of population			0.038		0.028	0.014
			(0.189)		(0.190)	(0.198)
Log of GDP per capita	0.255	-0.698*	0.318	-0.718*	0.305	0.301
	(0.210)	(0.368)	(0.707)	(0.369)	(0.709)	(0.714)
Constant	-1.414	7.618**	1.936	7.806**	2.289	2.661
	(2.068)	(3.502)	(4.795)	(3.512)	(4.881)	(5.043)
Observations	133	133	133	133	133	133
R^2	0.010	0.107	0.136	0.109	0.138	0.139
Adjusted R^2	0.003	0.093	0.102	0.095	0.104	0.098

Note: Robust standard errors in brackets. *p< 0.1 **p < 0.05 ***p<0.01.

Emission intensity: We explore whether digital products trade adds to the ability of economic complexity to explain emission intensity by modelling the logarithm of a country's yearly greenhouse gas emissions per unit of GDP (in kilotons of CO_2 equivalent per billion USD of GDP). Larger values represent larger emission intensity. For this analysis we use averaged data between 2016 and 2019. The baseline model includes the log of the GDP per capita (column 1 of Supplementary Table 5).

We find that physical trade complexity (ECI (physical)) is a significant and robust negative predictor of emission intensity (columns 2 and 3 of Supplementary Table 5). ECI (physical and digital) is also significantly and robustly related to emission intensity (columns 4 and 5 of

Supplementary Table 5). However, when we combine the two ECI in one regression model, both lose significance. This suggests that digital products trade complexity, for now, does not add significant new information about emission intensity.

Supplementary Table 5. Emission Intensity Regression Results.

	Dependent variable:					
	Log of GHG emissions per GDP (2016-19)					
	(1)	(2)	(3)	(4)	(5)	(6)
ECI (digital and physical)				-0.374***	-0.306***	0.267
				(0.066)	(0.074)	(0.545)
ECI (physical)		-0.373***	-0.305***			-0.568
		(0.065)	(0.073)			(0.535)
Log of exports per capita			-0.061		-0.061	-0.063
			(0.068)		(0.068)	(0.069)
Log of natural resource exports per capita			0.229***		0.230***	0.230***
			(0.056)		(0.057)	(0.057)
Log of population			0.063**		0.064**	0.062**
			(0.030)		(0.030)	(0.030)
Log of GDP per capita	-0.290***	-0.030	-0.262***	-0.028	-0.261***	-0.266***
	(0.045)	(0.057)	(0.090)	(0.058)	(0.091)	(0.092)
Constant	8.651***	6.180***	6.234***	6.157***	6.194***	6.301***
	(0.429)	(0.537)	(0.797)	(0.547)	(0.815)	(0.837)
Observations	137	137	137	137	137	137
R^2	0.273	0.427	0.480	0.425	0.478	0.480
Adjusted R^2	0.267	0.419	0.460	0.416	0.458	0.456

Note: Robust standard errors in brackets. *p< 0.1 **p < 0.05 ***p<0.01.

Supplementary references

1. Handbook on Measuring Digital Trade - OECD. https://www.oecd.org/sdd/its/handbook-on-measuring-digital-trade.htm.
2. Haberl, H. *et al.* A systematic review of the evidence on decoupling of GDP, resource use and GHG emissions, part II: synthesizing the insights. *Environmental research letters* **15**, 065003 (2020).
3. Hubacek, K., Chen, X., Feng, K., Wiedmann, T. & Shan, Y. Evidence of decoupling consumption-based CO2 emissions from economic growth. *Advances in Applied Energy* **4**, 100074 (2021).
4. Wang, Q. & Zhang, F. The effects of trade openness on decoupling carbon emissions from economic growth–evidence from 182 countries. *Journal of cleaner production* **279**, 123838 (2021).
5. Wiedenhofer, D. *et al.* A systematic review of the evidence on decoupling of GDP, resource use and GHG emissions, part I: bibliometric and conceptual mapping. *Environmental research letters* **15**, 063002 (2020).
6. Wu, Y., Zhu, Q. & Zhu, B. Comparisons of decoupling trends of global economic growth and energy consumption between developed and developing countries. *Energy Policy* **116**, 30–38 (2018).
7. Friedlingstein, P. *et al.* Global Carbon Budget 2022. *Earth System Science Data* **14**, 4811–4900 (2022).
8. Hidalgo, C. A. Economic complexity theory and applications. *Nature Reviews Physics* **3**, 92–113 (2021).

9. Balland, P.-A. *et al.* The new paradigm of economic complexity. *Research Policy* **51**, 104450 (2022).
10. Hidalgo, C. A. & Hausmann, R. The building blocks of economic complexity. *Proceedings of the national academy of sciences* **106**, 10570–10575 (2009).
11. Hartmann, D., Guevara, M. R., Jara-Figueroa, C., Aristarán, M. & Hidalgo, C. A. Linking economic complexity, institutions, and income inequality. *World development* **93**, 75–93 (2017).
12. Romero, J. P. & Gramkow, C. Economic complexity and greenhouse gas emissions. *World Development* **139**, 105317 (2021).
13. Stojkoski, V., Koch, P. & Hidalgo, C. A. Multidimensional economic complexity and inclusive green growth. *Communications Earth & Environment* **4**, 130 (2023).
14. Solow, R. M. A contribution to the theory of economic growth. *The quarterly journal of economics* **70**, 65–94 (1956).
